# Graphene–Oxide Porous Biopolymer Hybrids Enhance In Vitro Osteogenic Differentiation and Promote Ectopic Osteogenesis In Vivo

**DOI:** 10.3390/ijms23010491

**Published:** 2022-01-01

**Authors:** Aida Șelaru, Hildegard Herman, George Mihail Vlăsceanu, Sorina Dinescu, Sami Gharbia, Cornel Baltă, Marcel Roșu, Ciprian V. Mihali, Mariana Ioniță, Andrada Serafim, Horia Iovu, Anca Hermenean, Marieta Costache

**Affiliations:** 1 Department of Biochemistry and Molecular Biology, University of Bucharest, 91-95 Splaiul Independentei, 050095 Bucharest, Romania; aida.selaru@bio.unibuc.ro (A.Ș.); samithgh2@hotmail.com (S.G.); marieta.costache@bio.unibuc.ro (M.C.); 2 “Aurel Ardelean” Institute of Life Sciences, Vasile Goldis Western University of Arad, 86 Revolutiei, 310025 Arad, Romania; hildegard.i.herman@gmail.com (H.H.); baltacornel@gmail.com (C.B.); ramrosu@gmail.com (M.R.); mihaliciprian@yahoo.com (C.V.M.); 3 Faculty of Medical Engineering, University Politehnica of Bucharest, 1-7 Gh. Polizu, 011061 Bucharest, Romania; vlasceanu.georgemihail@yahoo.ro (G.M.V.); mariana.ionita@polimi.it (M.I.); 4 Advanced Polymer Materials Group, University Politehnica of Bucharest, 1-7 Gh. Polizu, 011061 Bucharest, Romania; andrada.serafim@gmail.com (A.S.); horia.iovu@upb.ro (H.I.); 5 Research Institute of the University of Bucharest, University of Bucharest, 050095 Bucharest, Romania; 6 Academy of Romanian Scientists, 54 Splaiul Independentei, 050094 Bucharest, Romania

**Keywords:** graphene oxide, biopolymer blends, biomineralization, ectopic bone formation, osteoinduction, ex vivo analysis

## Abstract

Over the years, natural-based scaffolds have presented impressive results for bone tissue engineering (BTE) application. Further, outstanding interactions have been observed during the interaction of graphene oxide (GO)-reinforced biomaterials with both specific cell cultures and injured bone during in vivo experimental conditions. This research hereby addresses the potential of fish gelatin/chitosan (GCs) hybrids reinforced with GO to support in vitro osteogenic differentiation and, further, to investigate its behavior when implanted ectopically. Standard GCs formulation was referenced against genipin (Gp) crosslinked blend and 0.5 wt.% additivated GO composite (GCsGp/GO 0.5 wt.%). Pre-osteoblasts were put in contact with these composites and induced to differentiate in vitro towards mature osteoblasts for 28 days. Specific bone makers were investigated by qPCR and immunolabeling. Next, CD1 mice models were used to assess de novo osteogenic potential by ectopic implantation in the subcutaneous dorsum pocket of the animals. After 4 weeks, alkaline phosphate (ALP) and calcium deposits together with collagen synthesis were investigated by biochemical analysis and histology, respectively. Further, ex vivo materials were studied after surgery regarding biomineralization and morphological changes by means of qualitative and quantitative methods. Furthermore, X-ray diffraction and Fourier-transform infrared spectroscopy underlined the newly fashioned material structuration by virtue of mineralized extracellular matrix. Specific bone markers determination stressed the osteogenic phenotype of the cells populating the material in vitro and successfully differentiated towards mature bone cells. In vivo results of specific histological staining assays highlighted collagen formation and calcium deposits, which were further validated by micro-CT. It was observed that the addition of 0.5 wt.% GO had an overall significant positive effect on both in vitro differentiation and in vivo bone cell recruitment in the subcutaneous region. These data support the GO bioactivity in osteogenesis mechanisms as being self-sufficient to elevate osteoblast differentiation and bone formation in ectopic sites while lacking the most common osteoinductive agents.

## 1. Introduction

The field of regenerative medicine and tissue engineering (TE) has emerged as a necessity for tissue substitutes in the case of major trauma. Therefore, the development of novel biomaterials to efficiently support tissue repair and regeneration is a serious matter in this research area. Thus, the goal of BTE relies on generating the expected support for the repair of bone defects based on biocompatible scaffolds with unique properties that enhance cell adhesion and formation of new bone extracellular matrix (bECM) and tissue.

High-performing artificial bone substitutes raise critical issues since engaging in the fabrication of autografts with equal performance to the “gold standard” is still technologically out of reach. Particularly in BTE, calcium (Ca^2+^) mineral-enriched scaffolds possess the ability to generate osteogenic signaling in osteoprogenitor populations [1]. Most mineral-based substrates investigated for these purposes successfully cover the mechanical features [2] but lack in the areas of morphological mimesis, pore interconnectivity, and pairing with organic compounds, unless they are supplied from allo-/xeno- graft sources. Even so, immunogenicity and unpredictable resorption rates can occur with low prospects of restraint [3]. 

Over the years, several polymers, mostly natural compounds, have been found to successfully mimic the bECM, thus generating a perfect microenvironment for cell proliferation, which is an important feature for BTE-designed scaffolds [4]. Fish gelatin, a derivate of a major component of the ECM, allures with good biocompatibility [5], high degradability rate [6] and low immunogenicity [7], while chitosan, due to its structural resemblance with glycosaminoglycans naturally sited in bECM, augments cell adhesion [8]. Therefore, gelatin and chitosan-based scaffolds have been demonstrated to meet the expected results in the case of osteogenic differentiation and engagement in bone repair [9,10]. Genipin is mostly used as a crosslinking agent for scaffold development due to its low toxicity and biosafety features [11,12,13]. Modern techniques in biomaterial design include the reinforcement of natural scaffolds with bioactive nanostructures. The oxygen-containing functional groups found in the GO structure ensure a great interaction with cellular proteins, hence significantly contributing to cellular behavior [14] in terms of cell growth and viability. GO has turned out to be a reliable nano-component due to its biocompatibility, which has been addressed in several studies over the years [15,16].

This carbon-based material turned out to present remarkable physiochemical characteristics, which have resulted in good biocompatibility and proper support for a plethora of next-generation targeted biomedical applications. Its versatility and tunable compatibility with robust and diverse materials captivated the focus in the research on skin [17] and adipose [18] regeneration, muscle (cardiac and skeletal [19]) engineering, nerve [20], as well as de novo cartilage and bone tissue [21]. GO demonstrated excitingly good interaction with many kinds of cell types, such as stem cells [22,23], neural cells [24,25], cardiomyocytes [26] and endothelial [27] cells and osteoblasts [28], while the non-toxic effect of GO-reinforced materials has been numerously reported in both in vitro and in vivo experimental conditions [29,30]. Even though there is not a global consensus on the drawbacks that GO additivities can be associated with, many results support that the impact of GO in the osteogenic development is rather positive, as long as the concentration of GO is not very high (~0.5 wt.%) [31,32,33].

Interestingly, studies have observed that the implantation of a scaffold engineered for BTE purposes at an ectopic site still has the means to recruit bone cells and to generate bone tissue, even if not surrounded by it [34]. Exquisite studies reported the implantation of BTE-designed scaffolds in other areas (e.g., subcutaneously/in muscle) in order to prove the osteoinductive and osteoconductive properties of the scaffolds [35,36,37,38]. These unique events have been proven for biomaterials such as β-tricalcium phosphate scaffolds [39], hydroxyapatite-based materials [40] and also phosphate graphene composites [41]. 

In our previous studies, we developed a new scaffold composed of fish gelatin/chitosan crosslinked with genipin (GCsGp) and reinforced with various GO:biopolymer mass ratios [15,42]. Appertaining on preliminary results, including thorough biocompatibility, we concurred and highlighted that the macromolecule network best performs if reinforced with 0.5 wt.% GO.

Predicated on these positive outcomes, we designed a study whose novelty is manifold: (i) complete characterization of partially investigated new BTE promising materials, (ii) survey of their specific osteoinductive features manifesting in ectopic sites and (iii) pioneering structural analysis of this kind of ex vivo sample. Firstly, we substantiated the materials’ characterization to the full extent, building on our previous findings on these types of composites. In this stage, we assessed their intrinsic osteoinductive properties in ectopic sites in mice models. Thus, by implantation in a non-osteogenic area, the number of variables involved in bone formations was reduced, eliminating the effects of bone stimulating cytokines, bone-forming cells and potentially bone-promoting mechano-transduction, and therefore, the onset of osteogenesis was attributed exclusively to the scaffold itself. 

Cell-laden hydrogels such as acid-g-chitosan-g-poly(*N*-isopropyl acrylamide) [43], bone morphogenetic protein-2 embedded collagen [44] or thiolated chitosan [45] exhibited promising bone formation when injected subcutaneously; furthermore, chitosan/calcium phosphate putties developed ectopic bone-like tissue when implanted intramuscularly.

To the best of our knowledge, our formulations are the only cell-barren polymer hybrids of this kind that manifest ectopic osteogenesis while lacking bone progenitor recruitment cues, differentiating inducers or Ca^2+^ and PO_4_^3−^ rich substrates. In addition, we showed that its remarkable behavior is strengthened by the presence of graphene oxide, known for synergistically promoting bone differentiation in hydroxyapatite composites [46] or as an osteomimetic support designed by phosphate functionalization of graphenic sheets [41].

Last but not least, we exploited these results from more than one perspective, and the paper advances a creative means to approach the explanted mineralized scaffolds from the standpoint of the engineer, issuing an uncommon process of physico-chemical characterizations meant to support and refine established immunohistological techniques.

## 2. Results and Discussion

The fish gelatin/chitosan crosslinked with genipin and reinforced with different GO biopolymer scaffolds have been previously analyzed by our group for their good physico-chemical properties and biocompatibility [15,42]. The in vitro osteogenic potential analysis and the de novo bone-forming capacity in vivo was compared for three materials: i. GCs (to evaluate the baseline osteoinductivity of an unmodified hybrid substrate); ii. GCsGp (to survey whether the osteoinductivity is conditioned by the biopolymer network crosslinking) and iii. GCsGp/GO 0.5 wt.% (to apprehend the contribution of GO to the de novo process of osteogenesis). These outcomes are supported by some studies which have focused on chitosan or gelatin-based materials enriched with graphene and its derivatives [22,47,48,49]. Thus, we used physical and morphological characterization of the material before implantation and on day 28 post-implantation in order to evaluate de novo osteoinductive properties of the materials by supplementation with GO in the absence of bone stimulating cytokines, bone-forming cells and potentially bone-stimulation mechano-transduction. The early stage of the osteogenesis under the material’s support was evaluated by using biological tests, respectively biochemical, histochemical methods and specific analysis of early and late markers of osteogenesis, both in vitro and in vivo.

### 2.1. A Priori Scaffold Characterization 

Engineered biomaterials for BTE can issue and propagate stimuli in cells regulating their early contact with a new lodging substrate, familiarization, adjustment and ultimately their phenotype outcome. This is most likely to occur due to the materials’ chemistry, physio-mechanical properties and distinctively tweaked nanostructuration [50].

**Figure 1:**
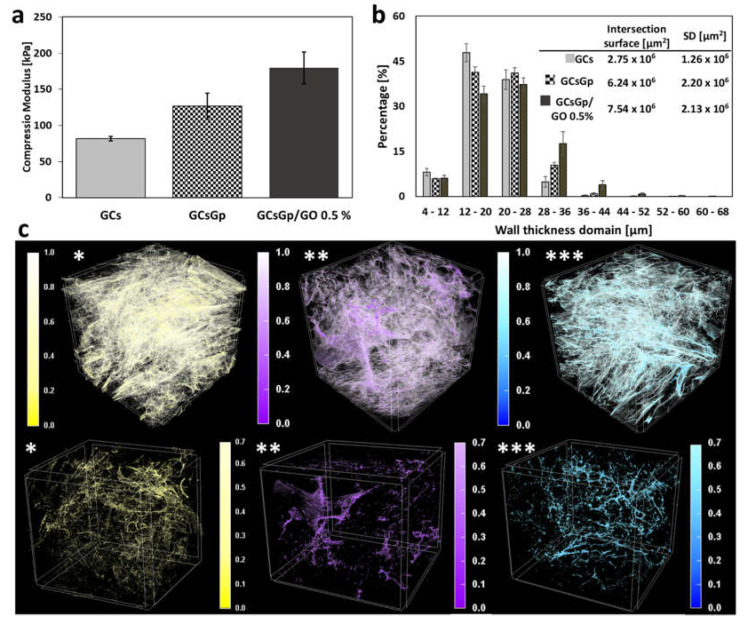
(**a**) Plotting of the compression modulus of hydrated materials, before implantation; (**b**) histogram depiction of the wall thickness size domain calculated in CTAn (Bruker); (**c**) color-highlighted 3D renderings of (*) GCs, (**) GCsGp and (***) GCsGp/GO 0.5% scaffold captured in CTVox.

Swollen freeze-dried GCs, GCsGp and GCsGp/GO 0.5% scaffolds, after reaching equilibria (2 h) [15], were subjected to mechanical testing meant to assess the effect of crosslinking and GO reinforcements generated within the GCs network with regards to compressibility. The measured values of E (plotted in Figure 1a as the average values ± SD) portray an expected image whereby the stiffness of the materials is augmented first by Gp crosslinking and additionally via GO embedding. In particular, compression modulus values are as following: E_GCs_ > E_GCsGp_ > E_GCsGp/GO 0.5%_ (81.67 kPa > 126.67 kPa > 179.50 kPa). 

**Figure 2:**
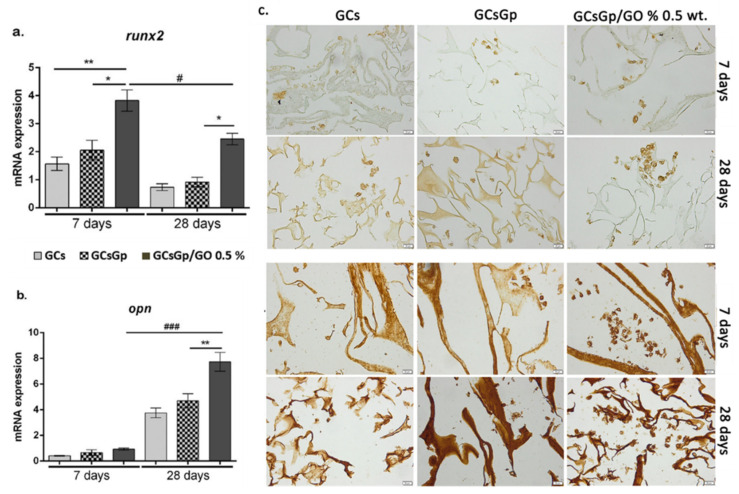
In vitro osteogenic profile analyses of runx2 (**a**) and opn (**b**) gene expression in differentiated 3T3-E1 cells in contact with GCsGp/GO materials with statistical significance ^###^ *p* < 0.001; ** *p* < 0.01; ^#,^* *p* < 0.05; (**c**) immunohistochemical runx2 and opn expression in differentiated 3T3-E1 cells in contact with GCsGp/GO materials.

The porous dry networks of the components were investigated by micro-CT analysis with the purpose of endorsing the mechanical behavior. Figure 2b consists of a chart of the incidence (in percentages) of dimensional domains as calculated for the scaffold walls. Gp, as well as consequent GO reinforcement, are able to customize the solid phase templating as a result of the GCs chains densification through crosslinking and additional centers of physical interactions supplied by the carbon nanomaterial within. As a consequence, scaffold walls tend to become thicker and stiffer—in agreement with the tendency of the detrimental shift the ratio of thinner walls exhibit with Gp and GO supplementation (Figure 1b). Moreover, the estimation of “intersection surface” values, areas of higher solid density where congruent walls meet, merge and overlap, support the theory according to which crosslinking and GO compositing of biopolymer blend favor the materialization of areas of variable density and stiffness. 

In order to provide a visual representation of the stiffness gradient, we depicted in Figure 1c a generic colored representation of the scaffold walls, pigmented in direct proportionality with the initial grey tones CT images possessed based on X-ray absorbance. Therefore, the areas where the colors are more pronounced feature superior agglomerations of solid matter (thicker walls), and the pale areas are associated with the finest layouts of the scaffolds. The classic greyscale (0–255) was converted in a reduced unit bar (0–1) for each colorized microtomography, and wall distribution was depicted both unaltered and 30% attenuated in the (0.7–1) region in order to highlight the volume spread of durotactic nuclei. 

In the case of GCs, they seem homogeneously spread within the sample volume, however light-consistent and sheer (Figure 1c *). GCsGp, on the other hand, features the coarsest associations of high-density domains but remotely distributed and preferentially toward the outer region of the scaffold (Figure 1c **). Antithetically, the GO composite (Figure 1c ***) displays a very interesting distribution of durotactic poles, with the highest isotropy in terms of both 3D distribution and dimensional extent. Consequently, its intrinsic structuration enables it to manifest the best mechanical performance in wet states, endorsed by synergistic Gp reticulation and GO embedding.

### 2.2. Effects of Graphene–Oxide Porous Biopolymer Hybrids on In Vitro Osteogenesis

In vitro osteogenic profile of GO-biopolymer composite resulted from experimental assay trials against 3T3-E1cell line. During the differentiation of pre-osteoblasts, Runx-related transcription factor 2 (runx2) is a master transcription factor, which is responsible for the regulation of other important osteoblast markers such as collagen type I alpha I (Col1a1) and osterix (SP7) [12]. In this study, qPCR evaluation of runx2 gene expression was evaluated after 7 and 28 days of osteogenic induction (Figure 2a) and revealed that murine pre-osteoblast differentiated successfully started the differentiation towards the osteogenic lineage. At 7 days, significant levels of runx2 expressions were found on the composites enriched with 0.5% wt.% GO as compared to the controls, GCs (*p* < 0.01) and GCsGp (*p* < 0.05). No significant differences were observed between the two tested controls, GCs and GCsGp, respectively. The expression on day 28 was found to be significantly (*p* < 0.05) lower as compared to the levels found after 7 days. This can be explained by the fact that runx2 acts in a stage-dependent manner during this process and is considered an early osteogenic marker, which is expected to present in higher expression levels within the first week of osteogenic differentiation. Moreover, it has been stated that the two isoforms of runx2, namely type I and type II, regulate different stages of a bone cell. Thus, runx2 type I is present in pre-osteoblasts [51], whereas runx2 type II is necessary for terminal phases of the osteogenic differentiation [52,53]. This comes in support of our results, which highlight that runx2 expression is still present after 28 days. Even so, the differences remained similar to those found at 7 days between the composites; namely, runx2 expression on GCsGp/GO 0.5% wt.% systems was found to be significantly higher (*p* < 0.05) as compared to GCs and GCsGp. 

At the same time, expression of the osteopontin (opn) gene (Figure 2b) was evaluated by qPCR. During osteogenesis, opn is highly expressed, as it produces an important protein, which is present in the bECM when cells achieve the stage of mature osteoblasts [31]. As compared to runx2, its levels of expression at 7 days of osteogenic differentiation were barely detectable as opn is a late osteogenic marker, which is expressed during the last weeks of osteogenesis.

Therefore, significant opn expression levels (*p* < 0.001) were found after 28 days of induction for the composite containing 0.5% wt.% GO in comparison to the expression levels found after 7 days of osteogenic induction (Figure 2b) on the same composite. Moreover, qPCR results for opn expression indicated a significantly increased (*p* < 0.01) expression on GCsGp/GO 0.5 wt.% in comparison to GCsGp control, after 28 days of osteogenic induction. As these results are in concordance with the one obtained in the case of runx2 expression, no significant opn expression levels were found on the two tested controls. Thus, these results suggest that incorporation of GO to GCsGp materials has significantly supported in vitro osteogenic differentiation of 3T3-E1 cells. A similar study has underlined that the addition of GO to composites based on poly(lactic-co-glycoid) acid surfaces [54] may enhance the expression of runx2 and opn during in vitro 3T3-E1 osteogenic differentiation. Moreover, it has been reported that in the presence of titanium surfaces with reduced GO [55], osteoblasts present a higher expression of opn bone marker.

A similar pattern was recorded for immunohistochemical expression of both RUNX2 and OPN osteogenic markers (Figure 2c). Here, it can be observed that the expression of both makers is present in the tested biosystems. The staining reveals the presence of differentiated cells within the pores of the material, by expression of RUNX2. Another study demonstrated that human mesenchymal stem cells have been found with a higher protein expression of RUNX2 in the presence of GO-collagen scaffold when compared to the collagen control [56]. The immunohistochemical staining for OPN expression showed a more pronounced expression for the composites enriched with GO, as compared to the GCs and GCsGp scaffolds. The expression of OPN and OCN by 3T3-E1 pre-osteoblasts has also been investigated by Lee et al. [46] in composites based on hydroxyapatite reinforced with reduced GO, where it was underscored that the presence of GO had an important contribution to the osteogenic differentiation of pre-osteoblasts. Moreover, cell clusters are predominantly present in the systems where GO was added, supporting once again the idea that the addition of GO has a beneficial impact on the cellular behavior during cell-scaffold interactions. These results come in support of the gene expression patterns found by qPCR, thus demonstrating the achievement of mature osteoblasts from 3T3-E1 precursors in contact with GO-enriched scaffolds.

**Figure 3:**
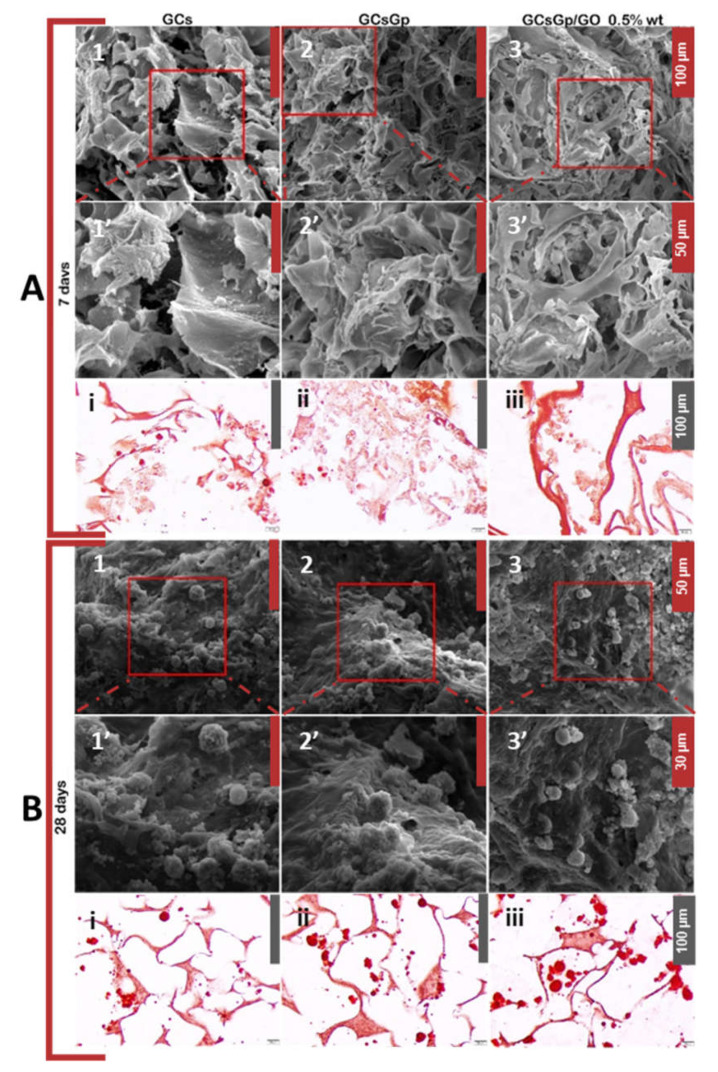
Qualitative evaluation of cellular distribution and morphology in GCsGp/GO scaffolds during 7 (**A1**–**A3**) and 28 (**B1**–**B3**) days of osteogenic differentiation using SEM while the **A1**’–**A3**’ and **B1**’–**B3**’ subsets depict corresponding close-ups of the areas marked in red squares above; qualitative evaluation of in vitro calcium accumulation in bECM using ARS histological staining at after 7 (**Ai**–**Aiii**) and 28 (**Bi**–**Biii**) days.

Cell morphology and distribution within the biocomposites were qualitatively evaluated by SEM. The obtained images revealed 7 days post-induction that cell adhesion occurred on all composites (Figure 3(A1–A3)). Interestingly, cells on GCsGp/GO 0.5% wt.% formed groups and populated the scaffolds’ pores. It can be observed that morphologically these cells present the characteristics of osteoblast precursors, namely a smooth surface structure with low amounts of mineral accumulation. 

After 28 days of in vitro differentiation, cell adhesion and spread are reconfirmed. It can be observed that cells exhibit different morphological features in opposition to those captured 7 days post-induction. In this latter case, it can be clearly distinguished that cells secreted a mineralized matrix on the surface and cells presented a cuboidal shape, thus demonstrating the presence of mature osteoblasts in the pores of the materials. 

ARS is a widely used histological staining to evaluate extracellular bone matrix accumulation and namely to qualitatively certify the observations on the SEM images. Seven days after osteogenic induction, only low amounts of calcium were detected in all scaffolds by ARS histological staining (Figure 3(Ai–Aiii)). Even so, these low quantities demonstrate the inception of the osteogenic differentiation in murine pre-osteoblasts. No significant differences were observed between the three tested composites within 7 days from induction. Twenty-eight days after induction, significant calcium accumulation can be observed in all the materials, in contrast to those found on day 7 (Figure 3(Bi–Biii)). It can be noticed that GO-enriched materials presented significantly more calcium aggregates in comparison to GCs and GCsGp scaffolds. Therefore, GO embedding in crosslinked GCs featuring the best pre-osteoblast differentiation motifs for in vitro osteogenesis is also confirmed by ARS staining.

Our previous studies [57,58,59] on GO-based composites underlined the active engagement of the 2D nanomaterial in cell adhesion and differentiation but also its welcome nature to catalyze cell viability and proliferation. Furthermore, we prove that functional GO-reinforced GCsGp scaffolds could serve as an osteoinductive matrix in vivo: seizing native cells to the site and harnessing differentiation into osteoblast in a non-osteogenetic area. We chose to study ectopic bone formation in the subcutaneous dorsal space of mice because osteoinductivity is clearly demonstrated, while the ability to develop bone in this non-specific location is more challenging and, thus, more persuasive of the innate properties of the scaffold [41,60,61].

### 2.3. Effects of Graphene–Oxide Porous Biopolymer Hybrids on Ectopic Bone Formation

In the in vivo study, we investigated the ectopic osteogenic differentiation potential of the scaffolds, without any advantage (dedicated cells, specific matrix, suitable growth factors) provided by an osteogenic area, wherein osteoblastic differentiation is stimulated by different signaling pathways (BMP, NF-kB, MAPK, Wnt) [62].

In vivo osteogenesis stimulated by GO addition in GCs networks was evaluated by quantitative analysis of biomarkers (seric ALP, opn and runx2) and the span of bECM deposits. Furthermore, by confocal microscopy, protein expression of opn and runx2 was illustrated, while histological staining results were captured under the light microscope. All of the mice survived until the retrieval of the implanted specimens, and there were no general or local complications.

**Figure 4:**
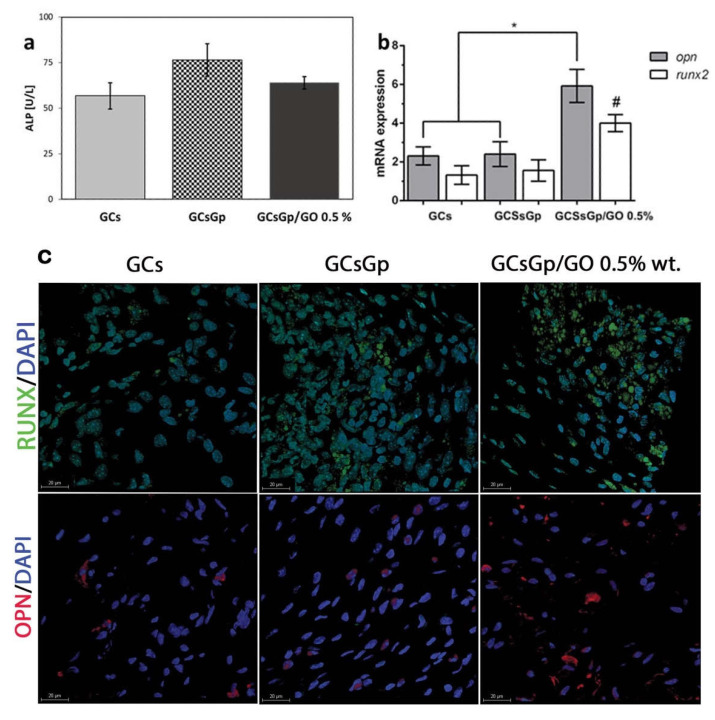
(**a**) Seric ALP activity 28 days post-implantation of GCs, GCsGp and GCsGp/GO 0.5% wt.% Scaffolds to mice; in vivo osteogenic profile analyses (**b**) mRNA expression of opn and runx2 four weeks post-implantation (statistical significance ^#,^* *p* < 0.05); (**c**) confocal microscopy protein expression of opn (red) and runx2 (green) and cell nuclei stained in blue four weeks post-implantation.

ALP is an early marker of osteogenic differentiation. The serum expression variations of ALP based on the nature of the scaffolds are not very high and borderline significant, but it still registers an increase with the addition of Gp, however, and is hindered by the presence of GO (Figure 4a). Of note, the presence of Gp as a crosslinking agent in the second tested control in this present study has channelled beneficial effects with respect to in vitro osteogenic differentiation, along the same lines with other studies that have explored the addition of this biomolecule in scaffolds designed for BTE [62,63].

The profile of osteogenic differentiation in implanted GCs, GCsGp and GCsGp/GO 0.5% wt.% was evaluated by qPCR analysis for two osteogenic markers, namely runx2 and opn. qPCR results indicated that 28 days post insertion, cells populated the material, since both osteogenic opn and runx2 genes were expressed in vivo (Figure 4b).

At the scaffold implantation site, the early osteogenesis process was quickly activated, showing an increased gene expression of the essential transcription factor runx2 toward Gp and Gp/GO enhanced scaffolds [64]. Furthermore, cell differentiation into mineralized matrix producing osteoblast phenotype was also stimulated by the GO enriched substrate during late osteogenesis, as shown by the immunopositivity of opn, which was strongly expressed 28 days after implantation. In a similar pattern, it was documented for runx2 and opn during osteoblast differentiation in vitro. 

After 28 days of in vivo state, both gene expressions were significantly lower in GCs and GcsGp compared to the GO-reinforced scaffold (*p* < 0.05). These data suggest the initiation of in vivo osteogenesis for all tested compositions, but with a significant stimulation per the scaffold containing 0.5% GO. Thus, these results indicate that the addition of GO brings an important contribution to the triggering and maturation of in vivo osteogenic differentiation. The confocal microscopy revealed the same pattern for opn and runx2 positivity (Figure 4c). It can be noticed that runx2 protein expression (green labeling) has been evidenced in the case of all three tested composites after 28 days of in vivo ectopic implantation, suggesting the presence of bone cells within the structure of the scaffold. Conversely, runx2 expression is stronger for all compositions as green staining fluorescence is visually clearest. It is better regulated in vivo rather than in vitro, advancing the idea that the master osteoblast regulator and transcription factor, promoter of key collagen I, ALP, opn and osteocalcin downstream genes [65], better intervene in support of osteoblast phenotype when in genuine physiological media. The obtained images indicate a more intense labeling in the case of a GCsGp/GO 0.5% system. The same can be stated in the case of opn protein expression (labeled in red), which has been found to be better outlined in the system where GO was added. opn, nonetheless, down-regulates osteoclasts cycles, is involved in bone matrix resorption, as it can bind calcium phosphates, and most likely connects superficial cell receptors to the support matrix through its specific RGD sequence [66]. For simply being expressed at the implantation site, one can analogize the resembling phenomena of the native bone metabolism cycle to the incipient event of ectopic bone formation within the implanted scaffolds.

**Figure 5:**
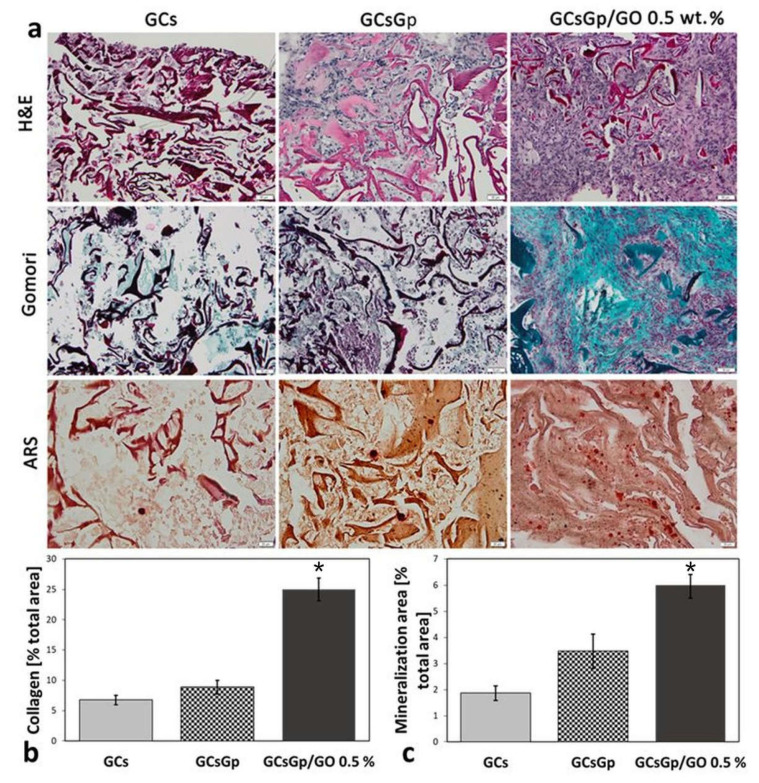
Histological analysis of the ectopic bone occurrence in GCs, GCsGp and GCsGp/GO 0.5% wt.% Scaffolds 28 days post-implantation. (**a**) Representative H&E, Gömöri trichrome and ARS stainings. Scale Bar 20 µm; (**b**) The analysis of the area of collagen domains according to Gömöri staining indicated that significantly more collagen was secreted within GCsGp/GO 0.5% wt.% Group as opposed to GCs group (* *p* < 0.001); (**c**). ARS staining indicates that significantly more calcium mineral deposits are present in the GCsGp/GO 0.5% wt.% group than GCs group (* *p* < 0.001).

Histological examination of the samples by H&E, Gömöri trichrome and ARS staining was performed under a light microscope, which enabled the detection of cell infiltration on all of the retrieved scaffolds implanted subcutaneously (Figure 5a). H&E staining demonstrated that the number of cells spread into the GCsGp/GO 0.5% network is superior to cell percolation observed against the controls. Furthermore, the highest amount of EC matrix found embedded in the scaffold pores corresponds to GO composite too. The GCs control, in particular, exhibits the poorest ECM penetration in the interconnected pore network; still, well-defined sectors were formed, preferentially following the durotaxis gradient (Figure 1c).

Gömöri’s trichrome staining was green positive in all samples, yet distinctly significant for GCsGp/GO 0.5% scaffold (*p* < 0.001). In this respect, collagen subsequently connected to create basic fibrous frameworks supporting bone formation. When reported to the bare GCs control, the production and in bulk pore occupancy by the secreted collagen was increased 3.7-fold upon GO and Gp supplementation and only by 1.2× for the scaffold reinforced with crosslinking agent alone (Figure 5b). Moreover, we assert that calcium mineral deposits developed against the foreign matrix of GCsGp/GO 0.5% and overlapped across the collagen Gömöri’s positive areas is a prompt but firm mark of ectopic bone formation. According to ARS staining, ectopic bone formation commenced mainly in GO-filled scaffold (*p* < 0.001 compared to GCs), while less evidence of mineralization could be identified in the two controls. In those cases, the nuclei of mineralization are rather disconnected or in the process of convergence (Figure 5c). 

The three staining assays concur in regards to the matter of the composition best endowed to support de novo bone tissue ingrowth. Twenty-eight days post-implantation, functional GO-reinforced 3D scaffold implants exhibited a better cellular infiltration and matrix production compared to GCs and GCsGp. Conformal to these intermediary staining outcomes, GO composites behaviorly outdo the in vivo performance of the controls. By its distinct nature, GCsGp/GO 0.5% excels at indulging the ectopic percolation of individual osteoblasts and finally the formation of the organic/inorganic osteogenic matrix.

GO embedded polymer-based frameworks for bone regeneration have been previously reported by us [59]. Our experimental results support the finding that GO promotes ectopic osteogenesis, as we have previously shown that with increasing graphene concentration in chitosan scaffolds implanted in bone defects, early and late osteogenesis marker expression is stimulated [57]. Other studies suggested innate osteoinductivity for GO, but the effects were weak under those conditions [19,67,68].

### 2.4. Dynamic Changes in Graphene–Oxide Porous Biopolymer Hybrids during Ectopic Bone Formation

Biomaterials, after explantation on day 28 after surgery, are only occasionally characterized from the morphological point of view and scarcely structurally. This lot, however, after retrieval, was subjected to some unconventional analyses to uncover possible clues pointing towards the certification of biochemical and immunohistochemical results. Firstly, µCT provided the global image of ectopic mineral formed inside each specimen, and customized image data analysis allowed a volumetric assessment of bone amount. By SEM, sharpened morphological aspects were provided, while some new bECM structuration theories were outlined empirically. FTIR and XRD spectra granted fundamental insight into the bi-phasic nature of the neotissue, from the angle of its patterning with respect to the remnant scaffold and the layout of the implant after long exposure to physiological media. In addition, structural analysis enables the corroboration of the osteoblast’s phenotype with the structuration of authentic bone by way of associating detected attributes of the explants to the acknowledged particularities of genuine tissue.

**Figure 6:**
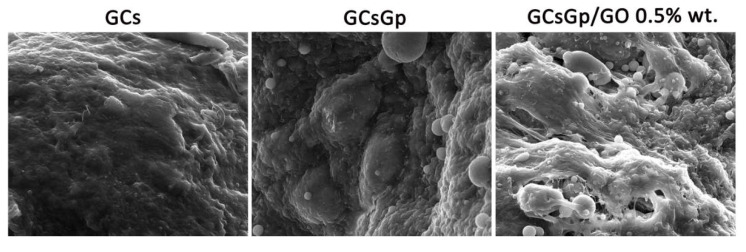
SEM micrographs of GCs, GCsGp and GCsGp/GO 0.5% wt.% scaffolds 28 days post-implantation.

**Figure 7:**
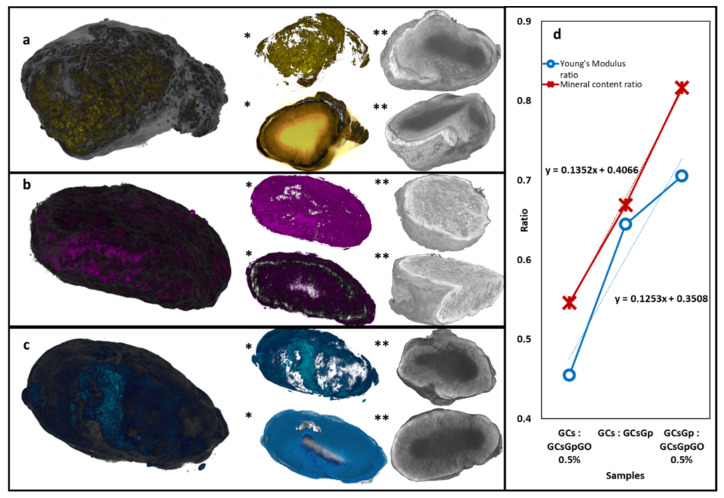
Colorized µCT images of (**a**) GCs, (**b**) GCsGp and (**c**) GCsGp/GO 0.5% wt.% scaffolds explanted 28 days; (*) marks indicate captures whereby the bi-phasic nature of the samples was separately highlighted and (**) marks indicate sectional views of the central morphology of the samples. (**d**) Charted data correlating mechanical properties and mineral formation based on the constitutional nature of the composites.

GO intrinsic osteoconductivity is also highlighted by the results obtained through SEM (Figure 6) and µCT (Figure 7) investigations. The SEM images of functional GO-reinforced GCsGp scaffolds showed a higher population of cell scaffolds with a differentiated phenotype towards osteoblasts and extensively secreted matrix, compared to GCsGp and GCs, respectively (Figure 6). On the pristine polymer scaffolds, bECM was formed in a lower amount and, based on the image contrast and surface texture, with a higher organic:inorganic ratio. The new collagenous deposits appear to be heavily laden with inorganic phase, most probably germinal calcium phosphates; still, it is not until the matter of GO composition that the phosphate phase of the ECM exceeds the organic quota. Furthermore, roughness is a heightened, topographical feature that favors the adhesion of circulating bone progenitor cells and the growth of tissue overall.

Moreover, these findings are supported by the E modulus measurement. E_GCsGp/GO 0.5_, despite being within the order of kPa while native bone sites reach 26 GPa [69], significantly favors the bone formation compared to the two control subjected in our study. GO, besides patterning the architecture of the 3D network [15], provides loci of amplified stiffness that delineates cell-friendly durotactic gradients [58]， in particular, beneficial for BTE [70]. 

µCT rendering showed that the biomineral deposits after 28 days of subcutaneous implantation reside preferentially on the outskirts of the scaffold, penetrating the volume to a lower extent. This phenomenon might be due to the fact that, initially, the recruited progenitor cells populate the interface of the living tissue with the artificial material. The proliferation within the volume can also be influenced by the size of the pores and the cell’s robustness to infiltrate through the interconnecting channels.

The resolution of the scan is 1.5 µm (pixel equivalent), so individual cells cannot be displayed; however, the aspect of the inorganic phase of the newly formed bECM suggests that tissue formation occurs in clusters casually spread at the interphase. These organizational domains feature a dense and compact aspect, as well as rough topography, resulted from the agglomeration of quasi-spherical phosphate deposits with slight irregularities. Incipient clusters of mineralization can also be identified in the innermost areas of the scaffolds, adhered to the resilient stiffer walls.

**Figure 8:**
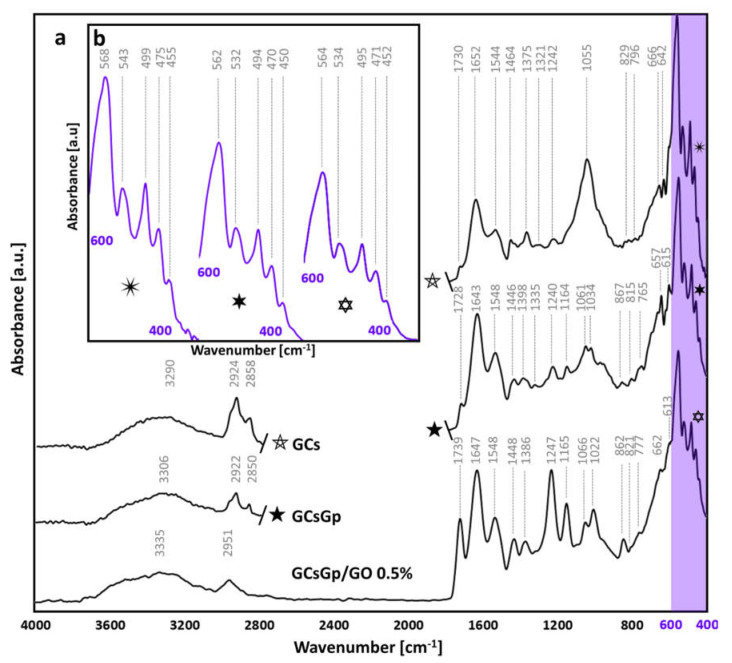
(**a**) FTIR spectra of GCs, GCsGp and GCsGp/GO 0.5% wt.% scaffolds explanted after 28 days; (**b**) close-up on the 400-600 cm^−1^ fingerprint domain specific to natural phosphates.

The 3D analysis enables the visualization of the dense crystalline phosphate agglomeration in light shades of gray and white while the lower density domains (original porous composite and collagenous share of the bECM) in darker tones. For a better visualization, in Figure 7a,a*,b,b*,c,c*, the mineral deposits were depicted in colors and the organic phase in white. Figure 7a**,b**,c** illustrates various angles and cross-sections of the tomograms without color alterations. Crystalline domains are contrastingly highlighted from the non-mineralized areas and delineate a gradient density sketch whereby the explant resembles a light-cored/dense-shelled model, as depicted in the cross-sectional views in Figure 8. Furthermore, the porous architecture of the materials is preserved in all compositions, even in the case of the uncrosslinked control. This remarkable stability might be due to the fact that the subcutaneous implantation inferred space constrictions, which limited the expected solvability. 

The CT datasets processing enabled the determination of organic/inorganic fractions in each composite, as cataloged in Table 1. Considering the scanning resolution, the total volume of the object was calculated by counting the three-dimensional pixel building blocks (voxel) of the tomograms and translating the voxel size to metric units; the object volume does not include the volume of the pores within. Mineral volume and non-mineral volume were determined by establishing a threshold level in the gray scale pallet of each tomogram, a clear separation of the inorganic/organic phases based on the image contrast. These quantitative data are detailed in Table 1, and the mineral percentage in each composition (vs. the object volume) is consistent with immunohistochemical and biomarkers assays and substantiates these findings. 

In addition, we plotted the mineral ratios of pairing composites against compression modulus ratios (Figure 7d) to survey the correspondence between the mechanics and osteogenesis, indicating linear and univocal proclivity between the two features. The durotaxis (cell guidance according to stiffness gradients) of GCs, GCsGp and GCsGp/GO 0.5% can be pinpointed by unified interpretation of compression test results and quantitative µCT data; the ratios between the E values and mineral formation follow a linear slope of direct proportionality. Durotaxis by itself confined the performance of the formulations (under both in vivo and in vitro angles) to linear variability. 

The nature of the dense crystalline domains in particular was further overviewed by FTIR and XRD. Figure 8 depicts the FTIR complex spectra of the explanted materials merging specific signal of both initial scaffolds and de novo material formation. The νOH broad domain of 3600–3000 cm^−1^ displays a shredded profile due to the plethora of local maximums of absorption connected to H bonding in remains of the initial implanted materials, the newly formed hybrid bECM and the interface of the two (Figure 8a). Furthermore, as some suggest, the domain can be seen as a heterogeneous area where proteic νNH signals (often ~3400 cm^−1^) [71] mingle with the νOH band [30]. Thoroughly addressing the convoluted band in similar scaffolding materials can be of remarkable interest in understanding the structural evolution of bioactive materials for BTE during the process of integration and regeneration. Symmetric and asymmetric stretching of the C–H bond appear within the 3000–2800 cm^−1^ range, indicating either specific to residues on the polysaccharide backbone [72] or lipidic tainting/formations in the bECM [73,74]; in GCs (2924 and 2858 cm^−1^) and GCsGp (2922 and 2850 cm^−1^), the maximum of absorption manifests slight shifts probably due to the important variations in the amount of new mineral formations and the molecular constraints that emerge as a result. In the GO composite, the symmetric stretching signal disappears as a broad domain in which a maximum of 2951 cm^−1^ is singularized.

Regarding protein structuration, amide I, II and III signals occur in all compositions. A signal at ~1730 cm^−1^ distinguishes with the Gp crosslinking and, consequently, with a GO addition that can be attributed to νC=O in amide I. Furthermore, the peaks in the range of 1700–1500 cm^−1^ are linked to amidic vibrations [75]. The amide II signal is weaker in GCs (1544 cm^−1^) due to the less chemically stable structure and incidental gelatin dissolution and increases with the control material’s crosslinking and compositing, manifesting a blue shift towards 1547 cm^−1^ [76]. Nonetheless, amide I fingerprint redshifts in GCsGp and GCsGp/GO 0.5% (towards 1643 and 1647 cm^−1^) [77]. In a similar fashion, the signals are stronger as a result of the collagen-based organic matrix formed in vivo and probably also as a result of the tendency of gelatin to renaturate to collagenic triple helical form. Amide III absorption peaks in the 1247–1240 cm^−1^ range [78]. GCs and GCsGp also exhibit weaker peaks at 1321 and 1375 cm^−1^ and 1335 and 1398 cm^−1^, which can be attributed to CO_2_ saturation of Ca^2+^ phosphates, whereas the GO composite features a stronger single peak at 1386 cm^−1^ [79,80]. Such variations indicate intimate interactions of mineral phases with the organic artificial matrix as well as the collagenous bECM. With respect to control, Gp and GO customization of the fish gelatin–chitosan hybrid generates material structurations that more resemble the natural bone molecular architecture [81]. Crosslinking on its own could partly renaturate the gelatin structure, while GO was shown to pattern the protein’s helicity closer to its natural state [82]. Moreover, the appearance of more defined peaks is supported by the protein rich content of the bECM secreted in the in vivo models. Initial matrix footprints emerge at ~1450 cm^−1^, where δCH_2_ signals lodge, related in particular to the proline ring [83].

In GCs, the strongest signal originates at 1055 cm^−1^ for the vibrations of νPO_4_^3−^ moiety in stoichiometric apatite formations [84]. After GCs crosslinking and GO embedding, its broadness is diminished and the peaks are split to 1061, 1034 and 1066, 1022 cm^−1^, respectively [85]. The lower wavenumbers emerging are indications of CO_3_^2−^ substitutions of Ca^2+^ and, thus, of fluctuations in crystallinity. The 1061/1034 and 1066/1022 ratios are flipped since the lower wavenumber absorbance is more enhanced in the case of GCsGp/Go 0.5% and still, within the domain of 1000–900 cm^−1^, particularly for the unsubstituted (crystalline) apatitic environment, a sharpening tendency is observed [85]. The decline in the crystallinity of in vivo biominerals is also partway supported by the 1164 cm^−1^ (in GCsGp) and 1165 cm^−1^ (GCsGp/Go 0.5%) peaks of less crystalline apatites; in addition, within the range of 900–750 cm^−1^, pyrophoshate specific signals emerge as markers of less ordered Ca^2+^ domains while above 600 cm^−1^, νOH vibrations from stoichiometric hydroxyapatite are highlighted. 

In the fingerprint region of 600–400 cm^−1^ (Figure 8b), the control (✴) features a very strong peak at 568 originating from PO_4_^3−^ ions, consistent with νPO_4_^3−^ vibrations in natural bones. For the crosslinked (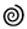) and GO inlayed composition (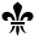), redshifts occur towards 562 and 564 cm^−1^, but not outside of the characteristics of ideal crystallinity of native bone structures [86,87]. Similarly, the position of the 475 peak (νPO_4_^3−^) in the control is pushed to lower wavenumbers. Intermediary vibrations were detected at 538 ± 5, 496 ± 3 and 452 ± 3 cm^−1^ assigned to δPO_4_^3−^ in apatites with various stoichiometrical coefficients [85,88].

**Figure 9:**
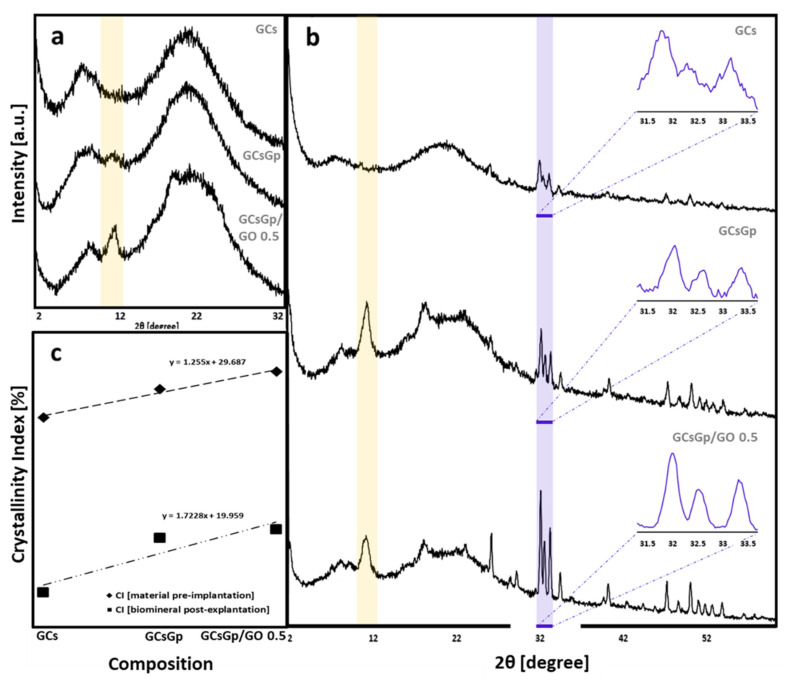
XRD spectra of GCs, GCsGp and GCsGp/GO 0.5% wt.% (**a**) before implantation and (**b**) after explanation; (**c**) crystallinity index of the three scaffold compositions.

The main target of XRD studies was to establish the influence of GO on GC composites’ general mode of structuration and ability to encourage osteoinduction by crystallinity index determination. According to the spectra displayed in Figure 9a, the XRD pattern of unloaded composite consists of two diffraction peaks located at 8.5° and 11.7°, one weak diffraction at 17.9° and a broad band around 21.5° [2]. The intensity maxima at 8.5° and 17.9° are assigned to the G domains (organized/unorganized). Their correspondent d-spacing values of 1.03 and 0.49 nm are in direct relation to the diameter of triple helical structures (lateral packing of gelatin) and to the isotropic amorphous region (the distance between amino-acid components), respectively [89,90]. The peak from 11.7° and the broadening domain at 21° ascribe to the semi-crystalline chitosan [91,92].

XRD spectra revealed that the addition of GO within the GCs matrix seems to cause alterations [2] within the unmodified samples spectrum, as the 11.7° peak associated with chitosan’s crystal I [93] and 21° band paired to the crystal II structure [94] significantly sharpen. Hence, it can be assumed that the identified sharpening is associated with a higher degree of crystallinity of the polysaccharide [95]; crystallization firstly occurred after crosslinking and accelerated with the GO embedding [30] within the matrix. This is confirmed by the crystallinity index (CI) that resulted from

CI = [(I_cr_ − I_am_)/I110] × 100, (1)

where I_cr_ is the maximum intensity of the diffraction peak of Cs, and I_am_ is the intensity of amorphous diffraction at 2θ = 16° [96]. CI values determined for GCs, GCsGp and GCsGp/GO 0.5% were: 30.84°, 32.4° and 33.55°, plotted in Figure 9c against the ones calculated for the ex vivo specimens.

G–Cs interactions partially result from hydrogen bonds and electrostatic interactions between carbonyl, amino and hydroxyl groups in polymer chains and genipin crosslinking. Generally, they facilitate the miscibility of the protein and polysaccharide but impede gelatin renaturation by decreasing the number of triple helices in the composite mass [97]. Nonetheless, by adding GO, gelatin renaturation reoccurs, as indicated by the 8.5° peak individualization.

The lack of GO signals within the FTIR spectra can be due to equipment limitations of detecting both the well dispersed and low amounts of GO sheets. Nevertheless, XRD characterization pointed out the fact that once GO sheets are incorporated within the hybrids, a slight sharpening of the maximum at 21° occurs, as well as an increase in intensity for the peak at 11.7° can be observed, suggesting that GO holds the ability to promote GCs crystalline features. With respect to the GCs pair affinity, the partial overlapping of the 17.9° peak with the 21° band is the result of crosslinking between the different species’ chains. The absence of GO specific intensity maximum from the composite’s spectra supports the idea of adequate GO nanosheet dispersion throughout the materials volume.

The XRD patterns of explanted scaffold (Figure 9b) reveal the tendency of the material to rearrange in more ordered patterns with Gp and moreover with GO addition. Explanted GCs exhibit a rather amorphous structuration with the exception of three sharper but weak peaks in the range of 31–34°. These signals appear in the more complex scaffolds too, featuring fewer broad extents and stronger intensities. Furthermore, the existence of sharp peaks, which generally characterize the hydroxyapatite at 25.9°, 28.9°, 31.9°/32.4°/32.0°, 32.3°/32.1°/32.0°, 33.0°/33.2°/33.2°, 46.7°, 49.8° and 53.2° corresponding to the diffraction planes (002), (120), (121), (112), (300), (222), (123) and (004) [98], respectively, indicate the presence of native-similar bECM in the explant structure. Meanwhile, the patterns of the crosslinked and composite matrices show two broad diffraction peaks centered at 2θ 11° and a broad band above 16°, which indicates their semi-crystalline nature. The peak identified at 11°, as well as the broad band are attributed to the semi-crystalline structure of chitosan and the unorganized and endorsed organized domains of gelatin. 

The XRD spectra suggest that overall crystallinity increases in the following order: GCs < GCsGp < GCsGp/GO 0.5%. The CI index calculated for the three specimens follow the same trend—linearly correlated with the CI of original scaffolds. This suggests that FTIR analysis cannot support crystallinity observations on its own; to reiterate, the FTIR spectra pointed out that upon customizing the control material, contrasting variations in stoichiometric and non-stoichiometric apatite vibrations were detected, with difficulty in providing a substantial judgment on the most ordered composition.

## 3. Materials and Methods

### 3.1. Scaffold Preparation

Graphene oxide powder, crab shell-derived medium molecular weight chitosan with 75–85% deacetylation degree, coldwater fish gelatin, genipin (purity > 98%—HPLC grade), and acetic acid (99.7%) were purchased from Sigma Aldrich (St. Louis, MO, USA) and used without prior purification. The composites’ synthesis was carried out in double distilled water.

Gelatin/chitosan (GCs), genipin crosslinked gelatin/chitosan blend (GCsGp) and 0.5 wt.% graphene oxide-reinforced genipin crosslinked gelatin/chitosan blend (GCsGp/GO 0.5%) scaffolds were prepared under identical conditions as previously reported [13]. Briefly, a GO dispersion procedure was carried out using a VCX 750 ultrasonic device from Sonics and Materials, Inc. (Newton, CT, USA) provided with a Ti-6Al-4V probe tip and a 750 W processor operating at 20 kHz. The amplitude of the probe tip vibrations was set at 70% throughout the 1 h GO exfoliation procedure. Gelatin was solubilized in water/GO aqueous dispersions (5% *w*/*v*) and mixed with the chitosan solution prepared in mild acidic solution (1% *v*/*v*). For a total of 50 mL solution, 8.33 mL of gelatin solution was homogenized with 41.67 mL chitosan solution. The crosslinking was carried out with genipin (1% *w*/*w*). Next, materials were frozen at −80 °C and freeze-dried (−55 °C).

### 3.2. Former Material Characterization

#### 3.2.1. Compression Tests

Compression tests were performed using a Brookfield CT3 texture analyzer equipped with a 4500 g cell. Freeze-dried samples with a diameter of 5 mm and a height of 3 mm were swollen at equilibrium, removed from the aqueous media and blotted dry before testing. The compressions were performed at a speed of 0.05 mm/s at room temperature. All measurements were performed in triplicate. A stress–strain graph was plotted using the dedicated software, and the compression modulus (E) was computed at 2% strain (in the linear part of the curve).

#### 3.2.2. Micro-Computed Tomography (µCT)

Freeze dried specimens of the GCs, GCsGp and GCsGp/GO 0.5% wt.% scaffold batch were scanned with Bruker µCT 1272 high-resolution equipment under the following conditions: no filter, 45 kV source voltage, 200 µA current intensity, 550 ms exposure per frame. The scanning was performed while samples rotated 180°, with a rotation step of 0.15. Every recorder projection was the averaged result of 6 acquisitions. Throughout the scaffold lot, the scanning resolution (image pixel size) was fixed at 4 µm. Tomogram reconstruction was performed in Bruker NRecon 1.7.1.6 software (Kontich, Belgium) and rendered in CTVox 3.3.0.0 (Bruker), while sample analysis was performed in CTAn 1.17.7.2 software (Bruker, Kontich, Belgium). For each composite, 4 cylindrical volume-of-interest (VOI) datasets (constrained in terms of diameter and height) were extracted. VOIs were subjected to an image-processing task list consisting of thresholding, despeckling, and 3D analysis (to quantify wall thickness and “intersection surface”). Wall thickness distribution was depicted while the values calculated for the intersection surface are tabulated in the adjacent inset (both with standard deviation ± SD).

### 3.3. In Vitro and In Vivo Biological Assessment

#### 3.3.1. In Vitro Differentiation of 3T3-E1 Cell Line in Contact with GCsGp/GO Biomaterials

Murine pre-osteoblasts from the MC 3T3-E1 cell line (ATCC) were seeded on GCs, GCsGp and GCsGp/GO composites at a density of 6.5 × 10^5^ cells/cm^2^ and incubated for 24 h in standard conditions (37 °C, 5% CO_2_ and humidity). Then, culture media was discharged and replaced with a commercially available osteogenic induction cocktail media (StemPro Osteogenesis Differentiation Kit, Thermo Fischer Scientific, Waltham, MA, USA). The osteogenic process was monitored for 28 days of in vitro cell culture and the differentiation media was changed every 3 days. The in vitro differentiation was evaluated at 7 and 28 days post-induction.

#### 3.3.2. Animals and Subcutaneous Mouse Model of Ectopic Bone Formation

CD1 male mice (6 weeks old, weight: 20–25 g) were used. Mice handling was carried out in accordance with the EU Directive 2010/63/EU and national legislation (Law No.43/2014). All experimental procedures have been approved by the Vasile Goldis Western University Ethics Committee for Research. Animals were housed in individually IVC cages, with ad libitum access to food/water, with standard conditions of temperature/relative humidity and a light/dark cycle of 12/12 h.

**Figure 10:**
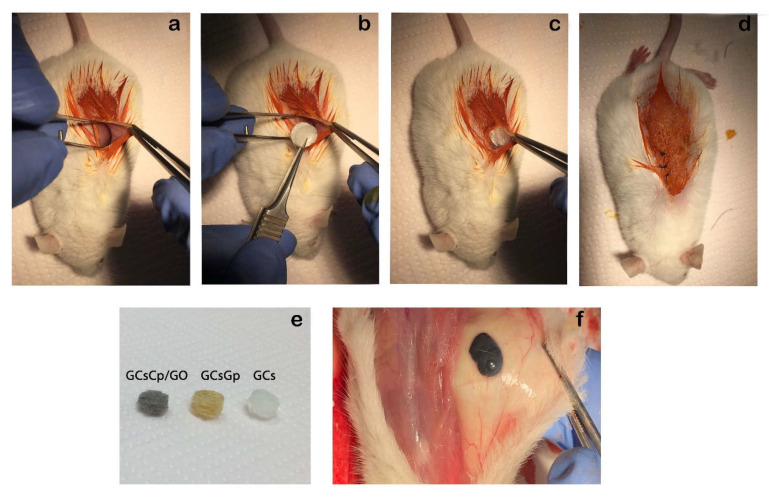
Experimental design. (**a**) Preparation of subcutaneous pocket in the dorsum of mice; (**b**,**c**) ectopic subcutaneous implantation of the scaffold; (**d**) closure of the overlaying skin; (**e**) scaffolds before implantation; (**f**) GCsGp/GO 0.5% wt.% scaffold 28 days after subcutaneously implantation to mice.

Surgical procedures were executed under anesthesia by intraperitoneal (i.p.) administration of 100 mg/kg b.w. ketamine hydrochloride and 10 mg/kg b.w. xylazine hydrochloride. Scaffolds were implanted ectopically into a subcutaneous pocket in the dorsum of the animals (Figure 10), randomly assigned to three groups (n = 10/group): 1 (GCs), 2 (GCsGp), 3 (GCsGp/GO 0.5 wt.%). After 28 days, mice were euthanatized, and the subcutaneous explants were removed and collected for further analysis. 

#### 3.3.3. Biochemistry

Blood samples were collected by cardiac puncture into sterile containers, without anticoagulant. Biochemical analysis was carried out to determine the serum level of alkaline phosphatase (ALP) using a biochemical analyzer (Mindray BS-120, ShenzenMindray Bio-Medical Electronics).

#### 3.3.4. Histology

The in vitro samples and the in vivo explants were fixed for 24 h in 4% paraformaldehyde, embedded in paraffin and cut in 5.0 μm thick sections. All samples were stained with Hematoxylin and Eosin (H&E) for morphological analysis and Alizarin Red S (ARS), to label calcium deposits as indicative of mineralization from cells displaying an osteogenic phenotype. Ex vivo explants were also stained by a Gömöri’s trichrome kit (Leica Biosystems) to demonstrate collagen synthesis. Microscopic sections were analyzed with an Olympus BX43 microscope.

#### 3.3.5. Immunohistochemistry

Immunohistochemical staining was performed on in vitro slides, with anti-mouse RUNX2 (diluted at 1:100; sc-390715, Santa Cruz Biotechnology, CA, USA) and OPN (diluted at 1:100; sc-73631, Santa Cruz Biotechnology, CA, USA) primary antibodies. For visualization, Novocastra Peroxidase/DAB kit (Leica Biosystems, Nussloch, Germany) was utilized, according to the manufacturers’ instructions.

#### 3.3.6. Immunofluorescence

The in vivo sections were incubated with primary antibodies against RUNX2 (diluted at 1:100; sc-390715, Santa Cruz Biotechnology, CA, USA) and OPN (diluted at 1:100; sc-73631, Santa Cruz Biotechnology, CA, USA), and then with secondary antibody conjugated with Alexa Fluor 488 flourescent dye (diluted at 1:200; A-11029, Thermo Fischer Scientific, Waltham, MA, USA). Finally, cell nuclei were visualized by DAPI and viewed under the confocal Leica TCS SP8 microscope system (Leica Biosystems, Nussloch, Germany).

#### 3.3.7. qPCR Analysis of Osteogenic Markers

Total RNA isolation was achieved by using TRIzol (Thermo Fisher Scientific, Waltham, MA, USA) and further RNA integrity number (RIN) was analyzed using an Agilent 2100 bioanalyzer. cDNA was synthesized using an iScript DNA synthesis kit (BioRad, Hercules, CA, USA) and was amplified by PCR using Veriti 96-well Thermal Cycler (Applied Biosystems, Waltham, MA, USA). qPCR was performed using SYBR Select Master Mix (Thermo Fisher Scientific, Waltham, MA, USA) and Viia7 equipment (Thermo Fisher Scientific, Waltham, MA, USA). Every sample was evaluated in triplicate and the gene expression of glyceraldehyde 3-phosphate dehydrogenase (GAPDH) was used as a reference gene (Table 2). 

#### 3.3.8. Statistical Analysis

The resulted data were statistically evaluated using one-way ANOVA method followed by a Bonferroni multiple comparison test. For this matter, GraphPad Prism 6.0 software (San Diego, CA, USA) for Windows 10 was used. All results are presented as mean ± SD of n = 3 experiments, and *p*-values < 0.05 were considered to be statistically significant. 

### 3.4. Ex Vivo Material Characterization (28 Days Post-Implantation)

#### 3.4.1. Fourier-Transform Infrared Spectrometry (FTIR)

FTIR investigations were carried on a SHIMADZU 8900 (Kyoto, Japan) on the subcutaneously explants, collected 28 days after material’s implantation, under Attenuated Total Reflectance (ATR) mode. The spectra resulted from the average of 32 acquisition with a resolution of 4 cm^−1^ over the range of 400–4000 cm^−1^.

#### 3.4.2. X-ray Diffraction (XRD)

X-ray diffraction measurements were performed at room temperature using a Panalytical X’Pert Pro MPD (Malvern, UK) instrument provided with a Cu Kα radiation source. For analogy reasons, XRD spectra were recorded before implantation and 28 days post-implantation.

#### 3.4.3. Scanning Electron Microscopy (SEM)

Both of the in vitro and in vivo samples were processed according to the technique described previously [33] and analyzed under scanning electron microscope—Quanta Inspect F SEM device equipped with a field emission gun (Fei Company, Hillsboro, OR, USA) with 1.2 nm resolution.

#### 3.4.4. Micro-Computed Tomography

Explated scaffolds were scanned with the same equipment as before the surgical procedure, under different parameters: 50 kV source voltage, 200 µA source current, 1200 ms exposure, 1.5 µm image pixel size. The rotation step was increased to 0.2° while the scan was performed upon a 360° sample rotation to avoid artifacts that may appear due to the presence of high-density mineral. Every projection was the averaged result of 3 acquisitions. Reconstruction, 3D illustration and bone mineral analysis were carried out in the same dedicated pieces of software provided by Bruker.

## 4. Conclusions

The aim of this study was to design a porous biopolymer hybrid as solutions to the lack of autologous bone needed to regenerate large defects in orthopedics. This is a pioneering account that graphene oxide incorporation in fish gelatin/chitosan/genipin scaffolds up-regulates both osteogenic differentiations in vitro and above all bone formation in ectopic sites when implanted in mice models.

To sum up, the data presented in this paper demonstrated that the addition of GO to the GCsGp composite enhances the expression of runx2 and opn during osteogenic differentiation. Moreover, data on collagen production and ectopic calcium deposits within the explanted composites originated from histology staining and underlined the capacity of the biomaterial alone to recruit bone cells in an ectopic site. Further, µCT results provided a measure of quality control over the Ca^2+^ biomineral survey. Overall, GCsGp/GO 0.5% wt was validated as the material with the strongest osteoinductive character, emphasizing, once again, the high gain from low GO supplementation of the polysaccharide-protein conjugate.

Bone formation within artificial materials is a process that is far from being fully comprehended down to its finely tuned mechanisms especially in the case of multi-component materials, which lack well-known bone-forming inducers and are structurally extremely complex. The present study covers the multi-angle investigation of a substrate of G and Cs, which we customized to a more bone-oriented scaffold by Gp crosslinking and GO embedding. These optimizations lead to a composite with osteoinduction-friendlier chemistry, crystallinity and durotaxis. The previously discussed results corroborate an overview understanding of material structuration and the GO-enabled features that best favor best the bone tissue formation in the experiment designed for this study. Quantitatively, the bECM secreted by the cells recruited to the implantation site was higher in the GO composite with the remark that the stoichiometry of in situ formed apatite was slightly below the GCs and GCsGp.

Among our perspectives, we consider addressing this issue upon a longer timeframe in order to gain insights on the osteoinductive manifestation of GCsGpGO materials and its aftereffects with respect to time. The variety of physical particularities and key chemical signals it provided enables the ranking of this survey as the first report on the osteogenic differentiation in vitro and bone formation in ectopic sites on the echelon of Ca^2+^ free GO embedded GCs blends.

## Figures and Tables

**Table 1:** Quantitative µCT measurements performed on the GCs, GCsGp and GCsGp/GO 0.5% samples.

Ex Vivo Sample	Object (Total) Volume (mm^3^)	Mineral Volume (mm^3^)	Non-Mineral Volume (mm^3^)	Mineral Percentage (%)
GCs	9.85	2.11	7.74	21.47
GCsGp	9.33	2.99	6.34	32.05
GCsGp/GO 0.5%	10.11	3.96	6.15	39.23

**Table 2:** List of primers used for qPCR analysis of osteogenic differentiation in 3T3-E1/GCsGp/GO biosystems.

Genes	Primers
*opn*	Forward: 5′-CTGGCAGCTCAGAGGAGAAG-3
	Reverse: 5′-TTCTGTGGCGCAAGGAGATT-3
*runx2*	Forward: 5′-ATCCCCATCCATCCACTCCA-3
	Reverse: 5′-GGGGTGTAGGTAAAGGTGGC-3′
*gapdh*	Forward: 5′-AACTTTGGCATTGTGGAAGG-3′ Reverse: 5′-ACACATTGGGGGTAGGAACA-3′

## References

[1] Nikolova M.P., Chavali M.S. (2019). Recent advances in biomaterials for 3D scaffolds: A review. Bioact. Mater..

[2] Foroutan S., Hashemian M., Khosravi M., Nejad M.G., Asefnejad A., Saber-Samandari S., Khandan A. (2021). A Porous Sodium Alginate-CaSiO 3 Polymer Reinforced with Graphene Nanosheet: Fabrication and Optimality Analysis. Fibers Polym..

[3] Di Silvio L., Jayakumar P., Di Silvo L. (2009). Cellular response to osteoinductive materials in orthopedic surgery. Cellular Response to Biomaterials.

[4] Filippi M., Born G., Chaaban M., Scherberich A. (2020). Natural polymeric scaffolds in bone regeneration. Front. Bioeng. Biotechnol..

[5] Şelaru A., Drăgușin D.-M., Olăreț E., Serafim A., Steinmüller-Nethl D., Vasile E., Iovu H., Stancu I.-C., Costache M., Dinescu S. (2019). Fabrication and Biocompatibility Evaluation of Nanodiamonds-Gelatin Electrospun Materials Designed for Prospective Tissue Regeneration Applications. Materials.

[6] Hayashi Y., Yamada S., Guchi K.Y., Koyama Z., Ikeda T. (2012). Chitosan and fish collagen as biomaterials for regenerative medicine. Adv. Food Nutr. Res..

[7] Lynn A.K., Yannas I.V., Bonfield W. (2004). Antigenicity and immunogenicity of collagen. J. Biomed. Mater. Res..

[8] LogithKumar R., KeshavNarayan A., Dhivya S., Chawla A., Saravanan S., Selvamurugan N. (2016). A review of chitosan and its derivatives in bone tissue engineering. Carbohydr. Polym..

[9] Ranganathan S., Balagangadharan K., Selvamurugan N. (2019). Chitosan and gelatin-based electrospun fibers for bone tissue engineering. Int. J. Biol. Macromol..

[10] Maji K., Dasgupta S., Pramanik K., Bissoyi A. (2016). Preparation and evaluation of gelatin-chitosan-nanobioglass 3D porous scaffold for bone tissue engineering. Int. J. Biomater..

[11] Dimida S., Barca A., Cancelli N., De Benedictis V., Raucci M.G., Demitri C. (2017). Effects of genipin concentration on cross-linked chitosan scaffolds for bone tissue engineering: Structural characterization and evidence of biocompatibility features. Int. J. Polym. Sci..

[12] Wang G., Zheng L., Zhao H., Miao J., Sun C., Ren N., Wang J., Liu H., Tao X. (2011). In vitro assessment of the differentiation potential of bone marrow-derived mesenchymal stem cells on genipin-chitosan conjugation scaffold with surface hydroxyapatite nanostructure for bone tissue engineering. Tissue Eng. Part A.

[13] Janfada A., Asefnejad A., Khorasani M.T., Joupari M.D. (2020). Reinforcement of electrospun polycaprolacton scaffold using KIT-6 to improve mechanical and biological performance. Polym. Test..

[14] Zhang B., Wei P., Zhou Z., Wei T. (2016). Interactions of graphene with mammalian cells: Molecular mechanisms and biomedical insights. Adv. Drug Deliv. Rev..

[15] Vlasceanu G.M., Șelaru A., Dinescu S., Balta C., Herman H., Gharbia S., Hermenean A., Ionita M., Costache M. (2020). Comprehensive appraisal of graphene–oxide ratio in porous biopolymer hybrids targeting bone-tissue regeneration. Nanomaterials.

[16] Patel S.K., Choi S.H., Kang Y.C., Lee J.K. (2017). Eco-friendly composite of Fe3O4-reduced graphene oxide particles for efficient enzyme immobilization. ACS Appl. Mater. Interfaces.

[17] Mahmoudi N., Eslahi N., Mehdipour A., Mohammadi M., Akbari M., Samadikuchaksaraei A., Simchi A. (2017). Temporary skin grafts based on hybrid graphene oxide-natural biopolymer nanofibers as effective wound healing substitutes: Preclinical and pathological studies in animal models. J. Mater. Sci. Mater. Med..

[18] Chu J., Shi P., Yan W., Fu J., Yang Z., He C., Deng X., Liu H. (2018). PEGylated graphene oxide-mediated quercetin-modified collagen hybrid scaffold for enhancement of MSCs differentiation potential and diabetic wound healing. Nanoscale.

[19] Bin Jo S., Erdenebileg U., Dashnyam K., Jin G.-Z., Cha J.-R., El-Fiqi A., Knowles J.C., Patel K.D., Lee H.-H., Lee J.-H. (2020). Nano-graphene oxide/polyurethane nanofibers: Mechanically flexible and myogenic stimulating matrix for skeletal tissue engineering. J. Tissue Eng..

[20] Saravanan S., Sareen N., Abu-El-Rub E., Ashour H., Sequiera G.L., Ammar H.I., Gopinath V., Shamaa A.A., Sayed S., Moudgil M. (2018). Graphene Oxide-Gold Nanosheets Containing Chitosan Scaffold Improves Ventricular Contractility and Function After Implantation into Infarcted Heart. Sci. Rep..

[21] Krukiewicz K., Putzer D., Stuendl N., Lohberger B., Awaja F. (2020). Enhanced Osteogenic Differentiation of Human Primary Mesenchymal Stem and Progenitor Cultures on Graphene Oxide/Poly(methyl methacrylate) Composite Scaffolds. Materials.

[22] Li M., Xiong P., Yan F., Li S., Ren C., Yin Z., Li A., Li H., Ji X., Zheng Y. (2018). An overview of graphene-based hydroxyapatite composites for orthopedic applications. Bioact. Mater..

[23] Kim J., Choi K.S., Kim Y., Lim K.-T., Seonwoo H., Park Y., Kim D.-H., Choung P.-H., Cho C.-S., Kim S.Y. (2013). Bioactive effects of graphene oxide cell culture substratum on structure and function of human adipose-derived stem cells. J. Biomed. Mater. Res. Part A.

[24] Bramini M., Alberini G., Colombo E., Chiacchiaretta M., DiFrancesco M.L., Maya-Vetencourt J.F., Maragliano L., Benfenati F., Cesca F. (2018). Interfacing graphene-based materials with neural cells. Front. Syst. Neurosci..

[25] Verre A.F., Faroni A., Iliut M., Silva C., Muryn C., Reid A.J., Vijayaraghavan A. (2018). Improving the glial differentiation of human Schwann-like adipose-derived stem cells with graphene oxide substrates. Interface Focus.

[26] Li X.P., Qu K.Y., Zhou B., Zhang F., Wang Y.Y., Abodunrin O.D., Zhu Z., Huang N.P. (2021). Electrical stimulation of neonatal rat cardiomyocytes using conductive polydopamine-reduced graphene oxide-hybrid hydrogels for constructing cardiac microtissues. Colloids Surf. B Biointerfaces.

[27] Mukherjee S., Sriram P., Barui A.K., Nethi S.K., Veeriah V., Chatterjee S., Suresh K.I., Patra C.R. (2015). Graphene oxides show angiogenic properties. Adv. Healthc. Mater..

[28] Di Carlo R., Di Crescenzo A., Pilato S., Ventrella A., Piattelli A., Recinella L., Chiavaroli A., Giordani S., Baldrighi M., Camisasca A. (2020). Osteoblastic Differentiation on Graphene Oxide-Functionalized Titanium Surfaces: An In Vitro Study. Nanomaterials.

[29] Maleki M., Zarezadeh R., Nouri M., Sadigh A.R., Pouremamali F., Asemi Z., Kafil H.S., Alemi F., Yousefi B. (2020). Graphene Oxide: A Promising Material for Regenerative Medicine and Tissue Engineering. Biomol. Concepts.

[30] Mollaqasem V.K., Asefnejad A., Nourani M.R., Goodarzi V., Kalaee M.R. (2020). Incorporation of graphene oxide and calcium phosphate in the PCL/PHBV core-shell nanofibers as bone tissue scaffold. J. Appl. Polym. Sci..

[31] Zeng Y., Zhou M., Chen L., Fang H., Liu S., Zhou C., Sun J., Wang Z. (2020). Alendronate loaded graphene oxide functionalized collagen sponge for the dual effects of osteogenesis and anti-osteoclastogenesis in osteoporotic rats. Bioact. Mater..

[32] Du Z., Wang C., Zhang R., Wang X., Li X. (2020). Applications of Graphene and Its Derivatives in Bone Repair: Advantages for Promoting Bone Formation and Providing Real-Time Detection, Challenges and Future Prospects. Int. J. Nanomed..

[33] Fang H., Luo C., Liu S., Zhou M., Zeng Y., Hou J., Chen L., Mou S., Sun J., Wang Z. (2020). A biocompatible vascularized graphene oxide (GO)-collagen chamber with osteoinductive and anti-fibrosis effects promotes bone regeneration in vivo. Theranostics.

[34] Du Z., Feng X., Cao G., She Z., Tan R., Aifantis K.E., Zhang R., Li X. (2020). The effect of carbon nanotubes on osteogenic functions of adipose-derived mesenchymal stem cells in vitro and bone formation in vivo compared with that of nano-hydroxyapatite and the possible mechanism. Bioact. Mater..

[35] Chai Y.C., Kerckhofs G., Roberts S.J., Van Bael S., Schepers E., Vleugels J., Luyten F.P., Schrooten J. (2012). Ectopic bone formation by 3D porous calcium phosphate-Ti6Al4V hybrids produced by perfusion electrodeposition. Biomaterials.

[36] Calabrese G., Giuffrida R., Forte S., Salvatorelli L., Fabbi C., Figallo E., Gulisano M., Parenti R., Magro G., Colarossi C. (2016). Bone augmentation after ectopic implantation of a cell-free collagen-hydroxyapatite scaffold in the mouse. Sci. Rep..

[37] Chen Z., Zhang Q., Li H., Wei Q., Zhao X., Chen F. (2020). Elastin-like polypeptide modified silk fibroin porous scaffold promotes osteochondral repair. Bioact. Mater..

[38] Karimi M., Asefnejad A., Aflaki D., Surendar A., Baharifar H., Saber-Samandari S., Khandan A., Khan A., Toghraie D. (2021). Fabrication of shapeless scaffolds reinforced with baghdadite-magnetite nanoparticles using a 3D printer and freeze-drying technique. J. Mater. Res. Technol..

[39] Ye X., Yin X., Yang D., Tan J., Liu G. (2012). Ectopic Bone Regeneration by Human Bone Marrow Mononucleated Cells, Undifferentiated and Osteogenically Differentiated Bone Marrow Mesenchymal Stem Cells in Beta-Tricalcium Phosphate Scaffolds. Tissue Eng. Part C Methods.

[40] Li W., Zheng Y., Zhao X., Ge Y., Chen T., Liu Y., Zhou Y. (2016). Osteoinductive Effects of Free and Immobilized Bone Forming Peptide-1 on Human Adipose-Derived Stem Cells. PLoS ONE.

[41] Arnold A.M., Holt B.D., Daneshmandi L., Laurencin C.T., Sydlik S.A. (2019). Phosphate graphene as an intrinsically osteoinductive scaffold for stem cell-driven bone regeneration. Proc. Natl. Acad. Sci. USA.

[42] Vlasceanu G.M., Crica L.E., Pandele A.M., Ionita M. (2020). Graphene oxide reinforcing genipin crosslinked chitosan-gelatin blend films. Coatings.

[43] Liao H.-T., Chen C.-T., Chen J.-P. (2011). Osteogenic Differentiation and Ectopic Bone Formation of Canine Bone Marrow-Derived Mesenchymal Stem Cells in Injectable Thermo-Responsive Polymer Hydrogel. Tissue Eng. Part C Methods.

[44] Bae I.-H., Jeong B.-C., Kook M.-S., Kim S.-H., Koh J.-T. (2013). Evaluation of a Thiolated Chitosan Scaffold for Local Delivery of BMP-2 for Osteogenic Differentiation and Ectopic Bone Formation. BioMed Res. Int..

[45] Zhang Q., He Q.-F., Zhang T.-H., Yu X.-L., Liu Q., Deng F.-L. (2012). Improvement in the delivery system of bone morphogenetic protein-2: A new approach to promote bone formation. Biomed. Mater..

[46] Lee J.H., Shin Y.C., Lee S.-M., Jin O.S., Kang S.H., Hong S.W., Jeong C.-M., Huh J.B., Han D.-W. (2015). Enhanced Osteogenesis by Reduced Graphene Oxide/Hydroxyapatite Nanocomposites. Sci. Rep..

[47] Mahanta A.K., Patel D.K., Maiti P. (2019). Nanohybrid Scaffold of Chitosan and Functionalized Graphene Oxide for Controlled Drug Delivery and Bone Regeneration. ACS Biomater. Sci. Eng..

[48] Wan C., Frydrych M., Chen B. (2011). Strong and bioactive gelatin–graphene oxide nanocomposites. Soft Matter.

[49] Depan D., Pesacreta T.C., Misra R.D.K. (2013). The synergistic effect of a hybrid graphene oxide–chitosan system and biomimetic mineralization on osteoblast functions. Biomater. Sci..

[50] Ventre M., Causa F., Netti P.A. (2012). Determinants of cell–material crosstalk at the interface: Towards engineering of cell instructive materials. J. R. Soc. Interface.

[51] Choi K.Y., Lee S.W., Park M.H., Bae Y.C., Shin H.I., Nam S., Kim Y.J., Kim H.J., Ryoo H.M. (2002). Spatio-temporal expression patterns of Runx2 isoforms in early skeletogenesis. Exp. Mol. Med..

[52] Xiao Z.S., Hjelmeland A.B., Quarles L.D. (2004). Selective deficiency of the “bone-related” Runx2-II unexpectedly preserves osteoblast-mediated skeletogenesis. J. Biol. Chem..

[53] Bruderer M., Richards R.G., Alini M., Stoddart M.J. (2014). Role and regulation of RUNX2 in osteogenesis. Eur. Cell Mater..

[54] Fu C., Yang X., Tan S., Song L. (2017). Enhancing cell proliferation and osteogenic differentiation of MC3T3-E1 pre-osteoblasts by BMP-2 delivery in graphene oxide-incorporated PLGA/HA biodegradable microcarriers. Sci. Rep..

[55] Liu M., Hao L., Huang Q., Zhao D., Li Q., Cai X. (2018). Tea polyphenol-reduced graphene oxide deposition on titanium surface enhances osteoblast bioactivity. J. Nanosci. Nanotehnol..

[56] Kang S., Park J.B., Lee T.J., Ryu S., Bhang S.H., La W.G., Noh M.K., Hong B.H., Kim B.S. (2015). Covalent conjugation of mechanically stiff graphene oxide flakes to three-dimensional collagen scaffolds for osteogenic differentiation of human mesenchymal stem cells. Carbon.

[57] Hermenean A., Codreanu A., Herman H., Balta C., Rosu M., Mihali C.V., Ivan A., Dinescu S., Ionita M., Costache M. (2017). Chitosan-graphene oxide 3D scaffolds as promising tools for bone regeneration in critical-size mouse calvarial defects. Sci. Rep..

[58] Ignat S.-R., Lazăr A.D., Şelaru A., Samoilă I., Vlăsceanu G.M., Ioniţă M., Radu E., Dinescu S., Costache M. (2019). Versatile Biomaterial Platform Enriched with Graphene Oxide and Carbon Nanotubes for Multiple Tissue Engineering Applications. Int. J. Mol. Sci..

[59] Dinescu S., Ionita M., Ignat S.-R., Costache M., Hermenean A. (2019). Graphene oxide enhances chitosan-based 3D scaffold properties for bone tissue engineering. Int. J. Mol. Sci..

[60] Salgado C.L., Teixeira B.I.B., Monteiro F.J.M. (2019). Biomimetic composite scaffold with phosphoserine signaling for bone tissue engineering application. Front. Bioeng. Biotechnol..

[61] Shabani I., Haddadi-Asl V., Soleimani M., Seyedjafari E., Hashemi S.M. (2014). Ion-exchange polymer nanofibers for enhanced osteogenic differentiation of stem cells and ectopic bone formation. ACS Appl. Mater. Interfaces.

[62] Osta B., Lavocat F., Eljaafari A., Miossec P. (2014). Effects of interleukin-17A on osteogenic differentiation of isolated human mesenchymal stem cells. Front. Immunol..

[63] Komori T. (2011). Signaling networks in RUNX2-dependent bone development. J. Cell. Biochem..

[64] Ling M., Huang P., Islam S., Heruth D.P., Li X., Zhang L.Q., Li D.-Y., Hu Z., Ye S.Q. (2017). Epigenetic regulation of Runx2 transcription and osteoblast differentiation by nicotinamide phosphoribosyltransferase. Cell Biosci..

[65] Choi J.-W., Shin S., Lee C.Y., Lee J., Seo H.-H., Lim S., Lee S., Kim I.-K., Lee H.-B., Kim S.W. (2017). Rapid Induction of Osteogenic Markers in Mesenchymal Stem Cells by Adipose-Derived Stromal Vascular Fraction Cells. Cell. Physiol. Biochem..

[66] Dubey N., Bentini R., Islam I., Cao T., Castro Neto A.H., Rosa V. (2015). Graphene: A versatile carbon-based material for bone tissue engineering. Stem Cells Int..

[67] Prasadh S., Suresh S., Wong R. (2018). Osteogenic potential of graphene in bone tissue engineering scaffolds. Materials.

[68] Shadjou N., Hasanzadeh M. (2016). Graphene and its nanostructure derivatives for use in bone tissue engineering: Recent advances. J. Biomed. Mater. Res. Part. A.

[69] Morgan E.F., Unnikrisnan G.U., Hussein A.I. (2018). Bone mechanical properties in healthy and diseased states. Annu. Rev. Biomed. Eng..

[70] Hadden W.J., Young J.L., Holle A., McFetridge M.L., Kim D.Y., Wijesinghe P., Taylor-Weiner H., Wen J.H., Lee A., Bieback K. (2017). Stem cell migration and mechanotransduction on linear stiffness gradient hydrogels. Proc. Natl. Acad. Sci. USA.

[71] Kourkoumelis N., Zhang X., Lin Z., Wang J. (2019). Fourier transform infrared spectroscopy of bone tissue: Bone quality assessment in preclinical and clinical applications of osteoporosis and fragility fracture. Clin. Rev. Bone Miner. Metab..

[72] Xu W., Wang W., Hao L., Zhao W., Liu H., Wang X. (2020). Effect of generation number on properties of fluoroalkyl-terminated hyperbranched polyurethane latexs and its films. J. Appl. Polym. Sci..

[73] Lui K., Jackson M., Sowa M.G., Ju H., Dixon I.M., Mantsch H.H. (1996). Modification of the extracellular matrix following myocardial infarction monitored by FTIR spectroscopy. Biochim. Biophys. Acta.

[74] Wang X.-F., Li M.-L., Fang Q.-Q., Zhao W.-Y., Lou D., Hu Y.-Y., Chen J., Wang X.-Z., Tan W.-Q. (2021). Flexible electrical stimulation device with Chitosan-Vaseline **^®^** dressing accelerates wound healing in diabetes. Bioact. Mater..

[75] Abasalta M., Asefnejad A., Khorasani M.T., Saadatabadi A.R. (2021). Fabrication of carboxymethyl chitosan/poly (ε-caprolactone)/doxorubicin/nickel ferrite core-shell fibers for controlled release of doxorubicin against breast cancer. Carbohydr. Polym..

[76] Liu H., Lin M., Liu X., Zhang Y., Luo Y., Pang Y., Chen H., Zhu D., Zhong X., Ma S. (2020). Doping bioactive elements into a collagen scaffold based on synchronous self-assembly/mineralization for bone tissue engineering. Bioact. Mater..

[77] de Campos Vidal B., Mello M.L.S. (2011). Collagen type I amide I band infrared spectroscopy. Micron.

[78] Unal M., Jung H., Akkus O. (2016). Novel Raman spectroscopic biomarkers indicate that postyield damage denatures bone’s collagen. J. Bone Miner. Res..

[79] Mata-Miranda M.M., Guerrero-Ruiz M., Gonzalez-Fuentes J.R., Hernandez-Toscano C.M., Garcia-Andino J.R., Sanchez-Brito M., Vazquez-Zapien G.J. (2019). Characterization of the biological fingerprint and identification of associated parameters in stress fractures by FTIR spectroscopy. Biomed. Res. Int..

[80] Smirnov I.V., Rau J.V., Fosca M., De Bonis A., Latini A., Teghil R., Kalita V.I., Fedotov A.Y., Gudkov S.V., Baranchikov A.E. (2017). Structural modification of titanium surface by octacalcium phosphate via Pulsed Laser Deposition and chemical treatment. Bioact. Mater..

[81] Imbert L., Gourion-Arsiquaud S., Villarreal-Ramirez E., Spevak L., Taleb H., van der Meulen M.C.H., Mendelsohn R., Boskey A.L. (2018). Dynamic structure and composition of bone investigated by nanoscale infrared spectroscopy. PLoS ONE.

[82] Ionita M., Crica L.E., Tiainen H., Haugen H.J., Vasile E., Dinescu S., Costache M., Iovu H. (2016). Gelatin–poly(vinyl alcohol) porous biocomposites reinforced with graphene oxide as biomaterials. J. Mater. Chem. B.

[83] Banc A., Desbat B., Cavagnat D. (2011). Ab initio calculations of proline vibrations with and without water: Consequences on the infrared spectra of proline-rich proteins. Appl. Spectrosc..

[84] Balan V., Mihai C.-T., Cojocaru F.-D., Uritu C.-M., Dodi G., Botezat D., Gardikiotis I. (2019). Vibrational spectroscopy fingerprinting in medicine: From molecular to clinical practice. Materials.

[85] Boskey A.L., Spevak L., Ma Y., Wang H., Bauer D.C., Black D.M., Schwartz A.V. (2018). Insights into the bisphosphonate holiday: A preliminary FTIRI study. Osteoporos. Int..

[86] Schuetz R., Fix D., Schade U., Aziz E.F., Timofeeva N., Weinkamer R., Masic A. (2015). Anisotropy in bone demineralization revealed by polarized far-IR spectroscopy. Molecules.

[87] Lopes C.D.C.A., Limirio P.H.J.O., Novais V.R., Dechichi P. (2018). Fourier transform infrared spectroscopy (FTIR) application chemical characterization of enamel, dentin and bone. Appl. Spectrosc. Rev..

[88] Woess C., Unterberger S.H., Roider C., Ritsch-Marte M., Pemberger N., Cemper-Kiesslich J., Hatzer-Grubwieser P., Parson W., Pallua J.D. (2017). Assessing various Infrared (IR) microscopic imaging techniques for post-mortem interval evaluation of human skeletal remains. PLoS ONE.

[89] Liu F., Antoniou J., Li Y., Ma J., Zhong F. (2015). Effect of sodium acetate and drying temperature on physicochemical and thermomechanical properties of gelatin films. Food Hydrocoll..

[90] Benbettaïeb N., Karbowiak T., Brachais C.-H., Debeaufort F. (2016). Impact of electron beam irradiation on fish gelatin film properties. Food Chem..

[91] Youssef A.M., Abou-Yousef H., El-Sayed S.M., Kamel S. (2015). Mechanical and antibacterial properties of novel high performance chitosan/nanocomposite films. Int. J. Biol. Macromol..

[92] Li Z., Yang F., Yang R. (2015). Synthesis and characterization of chitosan derivatives with dual-antibacterial functional groups. Int. J. Biol. Macromol..

[93] Naito P.K., Ogawa Y., Kimura S., Iwata T., Wada M. (2015). Crystal transition from hydrated chitosan and chitosan/monocarboxylic acid complex to anhydrous chitosan investigated by X-ray diffraction. J. Polym. Sci. B Polym. Phys..

[94] Jampafuang Y., Tongta A., Waiprib Y. (2019). Impact of crystalline structural differences between α-and β-chitosan on their nanoparticle formation via ionic gelation and superoxide radical scavenging activities. Polymers.

[95] Cheng J., Zhu H., Huang J., Zhao J., Yan B., Ma S., Zhang H., Fan D. (2020). The physicochemical properties of chitosan prepared by microwave heating. Food Sci. Nutr..

[96] Song X., Liu L., Wu X., Liu Y., Yuan J. (2021). Chitosan-Based Functional Films Integrated with Magnolol: Characterization, Antioxidant and Antimicrobial Activity and Pork Preservation. Int. J. Mol. Sci..

[97] Tronci G., Russell S.J., Wood D.J. (2013). Photo-active collagen systems with controlled triple helix architecture. J. Mater. Chem. B.

[98] El-Meliegy E., Abu-Elsaad N.I., El-Kady A.M., Ibrahim M.A. (2018). Improvement of physico-chemical properties of dextran-chitosan composite scaffolds by addition of nano-hydroxyapatite. Sci. Rep..

